# Auto-expansion software prompting reduces abbreviation use in electronic hospital discharge letters: an observational pre- and post-intervention study

**DOI:** 10.1186/s12911-025-03005-8

**Published:** 2025-05-01

**Authors:** Shamus Toomath, Emily J Hibbert

**Affiliations:** 1 Royal Prince Alfred Hospital, Camperdown, Sydney, Australia; 2 Nepean Clinical School, Faculty of Medicine and Health, The University of Sydney, Sydney, Australia; 3 Department of Endocrinology, Nepean Hospital, Sydney, Australia

**Keywords:** Electronic discharge letters, Auto-expansion software, Patient safety, Health communication, Medical abbreviations

## Abstract

**Background:**

Abbreviation use remains a significant cause of miscommunication among healthcare practitioners worldwide, creating uncertainty in interpretation and leading to poorer patient outcomes. This study aimed to assess the effectiveness of implementing auto-expansion prompts to reduce abbreviation use in electronic discharge letters (eDLs).

**Methods:**

Observational pre- and post-intervention study conducted in 2019 at a tertiary referral hospital in Western Sydney.

**Participants:**

Junior medical officers (JMOs) in postgraduate years 1 and 2.

**Intervention:**

The intervention consisted of an email invitation to JMOs, outlining the risks of abbreviation use in eDLs, and providing instructions on how to use auto-expand prompts for 11 commonly used abbreviations in *Cerner Powerchart*.

**Primary outcome measure:**

Reduction in the frequency of use of 11 commonly used abbreviations selected for auto-expansion, measured by a 200 eDL audit pre- and post-intervention.

**Secondary outcome measures:**

Reduction in the total number of abbreviations used and the mean number of abbreviations per eDL in the post-intervention audit compared to pre-intervention.

**Results:**

The baseline audit identified 1668 abbreviation uses in 200 eDLs, consisting of 350 different abbreviations. In the post-intervention audit, use of the 11 auto-expand abbreviations decreased by 43.6%, with decreased frequency of use for 9 of the 11 abbreviations. Post-intervention there was a 34.4% reduction in the total number of abbreviations used, with 1093 abbreviations identified in 200 eDLs.

**Conclusions:**

Advising JMOs to implement auto-expansion prompts for specific abbreviations, in combination with education on the risks of abbreviation use, is a cheap and effective solution to reducing abbreviation use in eDLs. This approach could significantly improve clarity of communication between hospital doctors and community healthcare professionals during patient care transition, potentially reducing medical errors.

## Background

Deficiencies in communication and information transfer between health care professionals (HCPs) lead to poorer outcomes and higher risks for patients [1–3]. Abbreviations have been directly implicated in medication prescription and administration errors [4, 5]. However, they are used frequently in medical records to save documentation time [2]. Although abbreviations have been a mainstay of medical communication for decades [6, 7], their use has been called into question due to high variability in both their intended meaning and understanding by others [1, 3, 8–10]. A 2011 Australian study revealed that only 43% of common abbreviations used in surgical inpatient admission notes were correctly interpreted by hospital multi-disciplinary team members [11]. A 2015 study in a Sydney tertiary hospital selected the 6 most-commonly-used abbreviations from electronic Discharge Letters (eDLs) and asked local General Practitioners (GPs) to interpret them in the context in which they had been used. The abbreviations were misinterpreted by up to 47% of GPs, demonstrating that abbreviations used in hospital eDLs are poorly understood by the GPs who receive them [1]. Another Australian study (2021) found that 99% of 802 hospital discharge letters (DLs) contained abbreviations. Worryingly, for 20 clinically relevant abbreviations, there was no consistent interpretation among 254 GPs and 62 Junior Medical Officers (JMOs) [12]. Similarly concerning, a significant number of these abbreviations could not be interpreted at all: 17.9% by GPs, 15.2% by JMOs. Furthermore, 94% of GPs worried about the potential adverse impact on patient care of using unclear abbreviations in DLs; 60% reported spending excessive time clarifying abbreviations [12]. Hospital DLs are critical for safe patient transfer of care and have potential to cause harm if they are unclear [1, 10].

Overuse of abbreviations and confusion around their meaning is a worldwide problem. In an Austrian retrospective audit of DLs, 750 unexplained abbreviations were found in 100 DLs [15]. In Israel, Shilo and Shilo found that medical and orthopaedic senior physicians were unfamiliar with the meaning of 14% and 25% of medical abbreviations, respectively, used in DLs by JMOs [13, 14]. Other international studies confirm these findings [16]. This has led to calls for a standardised list of abbreviations to be agreed upon and adhered to [11], or alternatively total avoidance or restriction of abbreviation use [13, 14, 17].

Education programs directed at doctors and other prescribers have been trialled to reduce the number of abbreviations being used in medical records [18, 19]. A Saudi Arabian study trialled an extensive education program for HCPs on the dangers of abbreviation use, followed by hospital-wide prohibition of their use, resulting in a 65% reduction in abbreviation use [19].

In recent years, attention has shifted to use of computer software to help prevent abbreviations making their way into medical records [3, 20–22]. Myers et al. assessed whether computerized alerts for unapproved medication abbreviations (eg.U, QD) in electronic medical progress notes could decrease unapproved medication abbreviation use in physicians’ hand written notes to reduce medication errors [20]. Fifty nine internal medicine interns were randomised to a ‘forced correction’ group, where they could not save or print a progress note until unapproved medication abbreviations were written in full, to autocorrection of the abbreviations or to a control group. Use of unapproved abbreviations in handwritten notes decreased in all groups, but most in the forced correction group [20]. Software trials aimed at retrospectively auto-expanding abbreviations in clinical records have reduced abbreviation use but also resulted in expansion errors [3, 22–24]. Accurately expanding abbreviations into their full terms remains difficult, even for advanced algorithms [3, 24].

A large retrospective audit of abbreviation use at Royal Melbourne Hospital [10] used automated software to identify abbreviations in 2336 hospital discharge letters. They identified 137,997 abbreviations. These abbreviations were then manually expanded by the authors [10]. Abbreviations were categorised as standardised (68.1%), largely pertaining to pathology/chemicals, or “non-standardised” (31.9%). The authors conclude that “auto-expansion of ambiguous abbreviations is likely to reduce miscommunication and improve patient safety” but acknowledged the limitations in retrospective expansion of abbreviations [10].

Thus, it remains clear that abbreviation use remains a significant cause of miscommunication among HCPs worldwide, creating uncertainty in interpretation and leading to poorer patient outcomes [1, 2, 24]. While computer software has shown promise in reducing abbreviation use, retrospective auto-expansion of abbreviations in pre-existing records has limitations, suggesting that auto-expansion would be best used at the time an HCP enters the abbreviation into the medical record [10, 24].

To the best of our knowledge, there are no published studies using auto-expansion prompts to reduce abbreviation use in hospital electronic discharge letters (eDLs) *at the time of eDL creation.* This study aimed to determine the incidence of abbreviation use in eDLs prior to and following introduction of an auto-expand software prompt for a limited number of abbreviations, to be used at the time of eDL creation in combination with brief email advice highlighting the dangers of abbreviation use in eDLs.

## Methods

This was a pre and post intervention observational study conducted at Nepean Hospital, a tertiary referral hospital in New South Wales.

### Baseline eDL audit

A retrospective baseline audit of 200 sequential eDLs was conducted moving forwards from 11th February 2019, using the method described by Chemali et al. [1]. Nepean Hospital Health Information Management Service generated the eDL list. eDLs came from the range of admitting specialties across the hospital. Abbreviations and their frequency were extracted from the body of each eDL, excluding other attached documents, such as imported lists of blood test results. Similar abbreviations e.g. BSL (Blood Sugar Level) and BGL (Blood Glucose Level) were combined, as were likely typographical errors such as HDSNM in place of HSDNM (heart sounds dual no murmurs).

### Development of the auto-expand prompt intervention

The 7 most commonly used abbreviations were determined from the baseline audit as well as 4 less commonly used abbreviations for which correct clinical interpretation was deemed by the investigators to be of high importance for optimal, safe patient care. These eleven abbreviations were then expanded into the full words or phrases intended using clinical interpretation from the context of their use, alongside reference to lists of commonly used abbreviations, a method used in similar studies [10]. The auto-expansion prompt intervention used pre-existing functionality in the electronic medical record software *Cerner Powerchart*, comprising a customisable dictionary that enables each user to add dictionary items for auto-expansion of specific abbreviations. Once an abbreviation was added to the auto-expansion dictionary, *Cerner Powerchart* prompted the user to auto-expand the abbreviation every time it was entered into the electronic medical record for that and for every subsequent patient record. Although the doctor was prompted to auto-expand the abbreviation, they could over-ride this if they continued to type the full word for the abbreviation themselves or moved onto the next word they wanted to type. In order to accept the auto-expansion they had to press ‘enter’ when the auto-expansion was offered as a prompt.

### Recruitment of junior medical officers for the study to implement the auto-expansion prompt intervention

Postgraduate year 1 (PGY1) and year 2 (PGY2) doctors were invited to participate in the study, as they write the majority of eDLs. Informed consent was obtained from the participants in the study. An invitation email was sent by the JMO Management Unit to all PGY1 and PGY2 doctors at Nepean Hospital. The email was sent twice. It highlighted the difficulty GPs have in interpreting abbreviations in eDLs and the risk this poses to patients. It invited JMOs to, with informed consent, implement auto-expansion prompts for eleven abbreviations (See Appendix A). The email had an attachment containing instructions on adding the eleven selected auto-expand abbreviations to JMO’s *Cerner Powerchart* dictionary accounts if they wished to participate.

### Post intervention audit of eDLs

Ten weeks after the first invitation email, a retrospective audit of 200 sequential discharge letters from Nepean Hospital was performed, moving forwards from 20th November, 2019.

### Outcome measures

The primary outcome was the change in incidence of use of the eleven abbreviations JMOs were asked to auto-expand from the baseline audit to post intervention. Secondary outcomes were the change in incidence of overall abbreviation use and in the number of different abbreviations used in eDLs from baseline to post intervention, the frequency of use of specific abbreviations and change in the mean number of abbreviations per eDL.

The study was approved by the NBMLHD Human Research Ethics Committee (2018/ETH00434).

## Results

### Baseline eDL audit

The majority of eDLs examined in the baseline audit were authored by postgraduate year 1 (PGY1) and year 2 (PGY2) doctors. There were 64 PGY1 doctors and 61 PGY2 doctors working across the Nepean Blue Mountains Local Health District in 2019, the majority based at the study hospital. A total of 1668 abbreviation uses were identified from the baseline 200 eDLs, comprising 350 separate abbreviations. The mean number of abbreviations per eDL was 8.5, with a range of 0 to 27. 78% of abbreviations identified were each used in five or fewer eDLs, and 86% were each used in ten or fewer eDLs. 49% of abbreviations were used just once.

Table 1 details the 35 most frequent abbreviations found in the baseline audit.

**Table 1:** Most-commonly used abbreviations from the baseline audit

Abbreviation	Frequency	Expansion
IV	73	Intravenous
ED	64	Emergency department
GP	44	General practitioner
PO	42	Per-oral
BD	40	Twice daily (Latin: bis in die)
PRN	37	When necessary (Latin: pro re nata)
CT	34	Computer tomography
PMHX	30	Past medical history
ABX	28	Antibiotics
GA	25	General anaesthetic
HPI	23	History of presenting illness
QID	22	Four times daily (Latin: quater in die)
EUC	21	Electrolytes urea creatinine
FU	21	Follow-up
HR	21	Heart rate
HTN	21	Hypertension
OE	21	On examination
ECG	20	Electrocardiogram
WCC	19	White cell count
SHX	19	Social history
CXR	19	Chest X-Ray
BP	19	Blood pressure
CRP	17	C-reactive protein
AF	17	Atrial fibrillation
MCS	17	Microscopy, culture and sensitivity
T2DM	16	Type 2 diabetes mellitus
DVT	17	Deep vein thrombosis
IVF	16	Intravenous fluid
TDS	15	Three times daily (Latin: ter die sumendum)
UA	14	Urinalysis
BG	14	Background
CTB	14	Computer tomography brain
LFTs	13	Liver function tests
CCF	13	Congestive cardiac failure

### Intervention

The eleven abbreviations chosen for inclusion in the auto-expansion prompt intervention are listed in Table 2. The first seven were the most commonly used abbreviations after exclusion of Latin abbreviations used in medication prescriptions, which are learned by all doctors, and the abbreviations IV, GP and CT, which were assumed to be understood by most doctors. The last four were chosen due to the clinical importance of understanding them. The combined invitation and intervention email was sent to all 126 JMOs at Nepean Hospital in PGY1 (64) and PGY2 (62).

**Table 2:** List of abbreviations included in the auto-expansion prompt intervention

Abbreviation	Expansion
PMHX	Past medical history
ABX	Antibiotics
GA	General anaesthetic
HPI	History of presenting illness
FU	Follow-up
HR	Heart rate
HTN	Hypertension
CCF	Congestive cardiac failure
COPD	Chronic obstructive pulmonary disease
IHD	Ischaemic heart disease
AKI	Acute kidney injury

### Post intervention eDL audit

A total of 1093 abbreviation uses were found in the 200 eDL post intervention audit. This was a 34.4% reduction in the number of abbreviation uses compared with the baseline audit. The mean number of abbreviations per eDL was 5.2, with a range of 0 to 17 abbreviations per eDL, a 41% reduction of 3.3 abbreviations per eDL from baseline. An additional 21 new abbreviations were identified that had not appeared in the baseline audit; however, the total number of different abbreviations decreased to 174, a 50% reduction.

For the eleven specific abbreviations for which auto-expansions had been recommended, there was a reduction in the frequency of use of 9 of the abbreviations post intervention. Incidence of the 11 abbreviations decreased by 43.6% from 259 in the baseline audit to 146 in the post intervention audit. However, there was an increase in the use of PMHx and no change in the use of HPI (Table 3).

**Table 3:** Frequency of abbreviations before and after the auto-expansion prompt intervention implementation

Abbreviation	Frequency, Pre-audit	Frequency, Post-audit
PMHX	30	39
ABX	28	23
GA	25	11
HPI	23	23
FU	21	4
HR	21	17
HTN	21	18
CCF	13	3
COPD	11	5
IHD	11	3
AKI	10	0

## Discussion

Our study found significant reductions in the incidence of abbreviation use in eDLs post intervention: a 43.6% decrease for the abbreviations we recommended JMOs auto-expand and a 34.4% decrease in overall incidence of abbreviation use. Furthermore, there was a 50% reduction in the number of different abbreviations used in the post-intervention audit. These decreases in abbreviation use are likely to be associated with improved eDL comprehension both by HCPs taking over patient care post discharge from hospital as well as amongst members of the multi-disciplinary team caring for hospital patients.

The results of this study are novel, in that it is the first study to our knowledge to significantly reduce abbreviation use in eDLs through a simple and cheap intervention, delivered by group email, combining brief education on the risks of abbreviation use and a strategy for reducing them through auto-expansion software. We hypothesize that the reduction in abbreviation use was related primarily to raising awareness of the potential harm of abbreviation use through the educational component of the invitation email. We believe that raising awareness resulted in a desire in junior doctors to reduce use of abbreviations. This seems to have been effective, as evidenced by a one third reduction in total incidence of abbreviation use, in addition to greater reductions in use of abbreviations we suggested be auto-expanded. Furthermore, the auto-expansion strategy, once set up in *Cerner Powerchart*, provided a time efficient method for reducing abbreviation use. Thus, it addressed the cause of abbreviation use, which is to save time [2].

Prior to the intervention, the mean number of abbreviations per eDL (8.7) was similar to that found in a 2015 audit conducted at the same hospital (7.1) [6]. These audits demonstrate the fact that, despite the Australian Commission on Safety and Quality in Healthcare’s 2017 guidelines urging doctors to minimize abbreviation use due to the potential for clinical safety risks [25], abbreviation usage remains high [12]. Efforts to reduce abbreviation use have thus far been largely ineffective, other than in the Saudi Arabian study which used a combination of an extensive education program for HCPs on the risk of abbreviations use followed by a hospital wide prohibition of use of abbreviations [19]. That study achieved a 65% reduction in abbreviation use. However, it is uncertain whether the intervention would be similarly effective in different contexts and cultures.

Since this study was conducted, *Cerner Powerchart* has acquired new functionality which will make it possible to share auto-expansions for abbreviations in the dictionary function across a group of healthcare professionals, such as all PGY1 and PGY2 doctors. This will remove the time barrier of JMOs needing to set up their own auto-expansions for abbreviations. Currently, JMOs receive several hours of training in medical record documentation using *Cerner Powerchart*. Training to implement the intervention used in this study requires only a few minutes more.

There are some important caveats to implementation of this type of software intervention effectively across a broad group of healthcare professionals. A glossary of agreed abbreviations is needed to standardize use of abbreviations at least across a facility but preferably nationally or internationally. It is essential that each abbreviation is mutually exclusive, so that there is a single auto-expansion for a single abbreviation. Not many abbreviations would require inclusion in this auto-expansion software, as in our study, as only 14% of abbreviations (49) were used over 10 times in 200 eDLs at baseline. This would need to be paired with a directive forbidding abbreviation usage outside the abbreviations in the auto-expand dictionary. In our study almost 80% of abbreviations were used in 5 or fewer eDLs, suggesting they may have been developed by individuals.

The main strengths of this pragmatic real- world study are the simplicity and negligible cost of the intervention, its efficacy in reducing the incidence of abbreviation use and its scalability. It is easily scalable across hospitals using *Cerner Powerchart*; for hospitals which do not use *Cerner*, there may be other software programs which can achieve the same outcome.

The major limitations of the study are that it was conducted at a single site and there was only a single post intervention audit, so we are unable to determine whether the effect of the intervention persisted or may have waned over time. If the effect waned, it is possible that it may be boosted by follow up emails delivered every few months. We cannot determine what proportion of the JMO cohort participated in the study, to what extent they used auto-expansion of abbreviations and to what extent they simply reduced their overall use of abbreviations. A potential limitation is the fact that the baseline audit was conducted on eDLs created early in the clinical year, when PGY1s are less experienced and the post intervention audit was conducted approximately 8 months later, so we cannot determine whether JMOs reduced abbreviation use as the year progressed, rather than due to the intervention. However, this seems unlikely as the previous audit by Chemali et al. [6] in the same hospital was conducted on eDLs produced late in the clinical year and found a similar mean number of abbreviations per eDL as we found in our baseline audit.

In conclusion, our study demonstrates that raising JMO awareness about the risk to patients of abbreviation use in eDLs and asking them to use auto-expansion software for abbreviations significantly decreases abbreviation usage. This represents a cheap, effective and scalable intervention for reducing abbreviation use and thus reducing health risks to patients during the transfer of care from hospitals to the community or other healthcare facilities. The results of this study are likely to be generalizable to hospitals using other software programs for creation of electronic medical records.

## Data Availability

No datasets were generated or analysed during the current study.
